# Psychophysiological Assessment of Children with Cerebral Palsy during Robotic-Assisted Gait Training through Infrared Imaging

**DOI:** 10.3390/ijerph192215224

**Published:** 2022-11-18

**Authors:** David Perpetuini, Emanuele Francesco Russo, Daniela Cardone, Roberta Palmieri, Chiara Filippini, Michele Tritto, Federica Pellicano, Grazia Pia De Santis, Raffaello Pellegrino, Rocco Salvatore Calabrò, Serena Filoni, Arcangelo Merla

**Affiliations:** 1 Department of Neuroscience and Imaging, University G. D’Annunzio of Chieti-Pescara, 66100 Chieti, Italy; 2 Padre Pio Foundation and Rehabilitation Centers, 71013 San Giovanni Rotondo, Italy; 3 Department of Engineering and Geology, University G. D’Annunzio of Chieti-Pescara, 65127 Pescara, Italy; 4 Department of Basic Medical Sciences, Neurosciences and Sense Organs, Institute of Child Neuropsychiatry, University of Bari, 70121 Bari, Italy; 5 Next2U s.r.l., 65127 Pescara, Italy; 6 Department of Scientific Research, Campus Ludes, Off-Campus Semmelweis University, 6912 Lugano, Switzerland; 7 IRCCS Centro Neurolesi “Bonino-Pulejo”, 98124 Messina, Italy; 8 ITAB, Institute for Advanced Biomedical Technologies, 66100 Chieti, Italy

**Keywords:** infrared thermography, cerebral palsy, robotic-assisted gait training, neurorehabilitation, rehabilitation, psychophysiological assessment, therapeutic engagement

## Abstract

Cerebral palsy (CP) is a non-progressive neurologic pathology representing a leading cause of spasticity and concerning gait impairments in children. Robotic-assisted gait training (RAGT) is widely employed to treat this pathology to improve children’s gait pattern. Importantly, the effectiveness of the therapy is strictly related to the engagement of the patient in the rehabilitation process, which depends on his/her psychophysiological state. The aim of the study is to evaluate the psychophysiological condition of children with CP during RAGT through infrared thermography (IRT), which was acquired during three sessions in one month. A repeated measure ANOVA was performed (i.e., mean value, standard deviation, and sample entropy) extracted from the temperature time course collected over the nose and corrugator, which are known to be indicative of the psychophysiological state of the individual. Concerning the corrugator, significant differences were found for the sample entropy (F (1.477, 5.907) = 6.888; *p* = 0.033) and for the mean value (F (1.425, 5.7) = 5.88; *p* = 0.047). Regarding the nose tip, the sample entropy showed significant differences (F (1.134, 4.536) = 11.5; *p* = 0.041). The findings from this study suggests that this approach can be used to evaluate in a contactless manner the psychophysiological condition of the children with CP during RAGT, allowing to monitor their engagement to the therapy, increasing the benefits of the treatment.

## 1. Introduction

Cerebral palsy (CP) is a non-progressive neurologic condition due to a brain injury that occurs before the complete cerebral development [1], representing a primary cause of motor disability in children. CP patients often exhibit muscle weakness, spasticity, bone deformities, impaired balance, and limited coordination. In addition, children with CP are often characterized by a decreased walking speed, an increased double support duration, and poor endurance, highly impacting their daily activities, quality of life, and social integration [1]. The incidence of this disease is estimated to be 2.08 per 1000 live births, but it increases 70 times when referring only to children born with a body weight below 1500 g [2].

Among the several clinical scales employed to evaluate the severity of motor disorders of CP, the Gross Motor Function Classification System (GMFCS) is one of the most commonly employed in clinical practice [3]. This scale investigates the child’s independence during basic motor functions, delivering a five class classification of the patients, where in class 1 individuals with full motor abilities are included, whereas in class 5 patients who are not able to move on their own are comprised [3].

To enhance the quality of life and social integration of CP patients, several pharmaceutical strategies [4] and rehabilitative procedures have been developed [5]. Robotic-assisted gait training (RAGT) is now a popular rehabilitation technique used to help patients with neurological problems improve their gait [6].

In fact, it was proved that the spinal cord’s central pattern generators (CPG), or gait centers, could be activated by passive motions of the limbs [7]. Moreover, it was demonstrated that patients with serious spinal cord injuries could also produce equivalent locomotor activity by passively activating their legs on a treadmill [6]. RAGT provides numerous advantages over traditional body weight support treadmill training methods. By giving more intense training than typical rehabilitation, RAGT, for instance, enables the early starting of rehabilitation even in severe cases. Additionally, RAGT compels patients to walk in more physiological and repeatable gait patterns, allowing for the evaluation of the patients’ performances. Finally, RAGT has positive effects on cardiopulmonary functions as well [8].

End-effectors and exoskeletons are the two types of RAGT devices that are readily accessible on the market. End-effectors essentially consist of a double crank and rocker gear system to exert forces on the distal segments of limbs, and a servo-controlled motor guides the patient during walking. Exoskeleton-type devices direct the patient’s legs according to a preprogrammed gait pattern using a robotic orthosis coupled with a harness-supported body weight system coupled with a treadmill.

The Lokomat^®^ (Hocoma AG, Volketswil, Switzerland) is one of the commercially available exoskeletons that supports the patient on a treadmill while a robotic device helps with inter-limb coordination and gait timing [9]. The system offers varying levels of body weight support and guidance that may be adjusted according to the patient’s requirements, making it particularly ideal and successful for treating CP in children [10]. In fact, it was shown that using the pediatric orthosis of the equipment could considerably reduce muscular stiffness following just one RAGT session [10].

In comparison to the traditional body weight support treadmill training methods, RAGT can result in a higher level of spine and brain neuroplasticity [6]. The central nervous system’s (CNS) capacity for long-term structural and functional adaptation in response to both internal and external stimuli is known as neuroplasticity [11]. The damaged brain can use this ability to change its capabilities in response to therapy. In particular, the neuroplasticity mechanism induced by rehabilitation may be able to help CP sufferers’ brains regain the ability to control their motor activities. Because the CNS is more pliable at the beginning of its development, early intervention for children with CP is important [5,12]. Hence, monitoring brain activity during the RAGT could reveal information on how the therapy affects neuroplasticity. To this aim, portable neuroimaging techniques, such as functional near infrared spectroscopy (fNIRS) and electroencephalography (EEG) are highly suitable, since they do not physically restrain the patients during the gait [13,14].

However, it should be highlighted that the effectiveness of the therapy is strictly related also to the engagement of the patient in the treatment. It has been suggested that the concept of “rehabilitation engagement” encompasses a number of factors, including the patient’s attitude toward therapy, their level of understanding or acknowledgment of a need for treatment, their requirement for verbal or physical prompts to participate, their level of active participation in therapy activities, and their level of attendance throughout the rehabilitation program [15]. Greater rehabilitative participation is linked to lower levels of depression, denial of illness, and negative affective states as well as higher levels of positive affective states. Additionally, conceptualizations and models of engagement in medical therapy have been proposed, including interest, motivation, persistence, and effort in rehabilitation therapies. Hence, monitoring the psychophysiological state and the activity of the autonomic nervous system (ANS) of the patients during the RAGT could provide information regarding their engagement to the rehabilitation treatment.

ANS activity could be inferred through the evaluation of the heart rate variability (HRV) and through electrodermal activity (EDA). Heart rate variability (HRV) is the fluctuation in the time between successive heartbeats that is primarily influenced by the control of heart rate (HR). The status of the ANS and overall cardiac health can be evaluated through HRV analysis [16,17,18]. Particularly, the HR is controlled by the balance between the sympathetic nervous system (SNS) and parasympathetic nervous system (PNS): dominance of the SNS activity with respect to that of the PNS produces an acceleration of the cardiac rhythm, whereas a prevalence of the PNS activation causes a deceleration of the HR. HRV is generated by the interaction between the heart and dynamic nonlinear ANS processes. As a result, the HRV reveals how well the autonomic nervous system regulates cardiac rhythm and how responsive the heart is [19]. Compared to children with normal development, pediatric patients with CP were demonstrated to show considerably greater resting heart rates, lower heart rate variability (HRV), and different autonomic responses to movement stimuli [20].

EDA measures skin conductance, which changes depending on the activity of the skin’s sweat glands. Skin conductance is a sign of psychological or physiological arousal since sweating is regulated by the sympathetic nervous system [21]. Sweating gland activity increases with increased sympathetic nervous system activity, which in turn raises skin conductance. So far, EDA was employed to assess CP children’s autonomic activity during virtual reality tasks [22,23].

However, positioning sensors on palmar surfaces is not recommended when recordings are likely to contain motion and pressure anomalies. Notably, when a human subject exhibits uncontrollable movements, such as in hyperkinetic CP, these artifacts are common [24].

To overcome this issue, the employment of contactless sensors able to infer human psychophysiological condition, such as infrared thermography (IRT), could be preferable since they do not need a preparation of the patients and they are completely non-invasive and comfortable also for long-term monitoring. IRT is able to measure the superficial temperature of a body at a distance. In the biomedical field, this technique is employed to measure the modulation of skin temperature, which is an indicator of a subject’s psychophysiological condition [25]. In fact, stress, anxiety, or weariness, but also happiness, may cause changes in superficial microcirculation that induces fluctuations in skin temperature. It is worth highlighting that superficial microcirculation could be assessed by employing several technologies. For instance, laser doppler flowmetry is a technique that allows to continuously and non-invasively monitor changes in microvascular perfusion with regard to the relative changes in blood volume and velocity [26]. Alternatively, photoplethysmography (PPG) is an optical technique able to measure the blood volumetric oscillations in vessels related to heart rate [27,28,29]. However, IRT is advantageous with respect to these techniques since they often require contact sensors that could be uncomfortable for long-term monitoring. In addition, although contactless PPG is commonly employed, IRT has the advantage of providing physiological information related to other processes such as breathing and sweat gland activity.

Hence, IRT has already been used to investigate the workload [30], the learning process [31], and the subject’s emotional state [25,32,33]. Furthermore, this technology has been used to assess autonomic correlates during the administration of clinical tests, for instance for Alzheimer’s disease patients [34] or during the recovery from breast cancer treatment [35].

The aim of this study is to assess the psychophysiological condition of children affected by CP during RAGT through IRT. To the best of the authors’ knowledge, this study is the first attempt to investigate the psychophysiological condition of CP patients during the RAGT, in order to provide information regarding the engagement of the children in the therapy.

## 2. Materials and Methods

### 2.1. Particpants

The participants were enrolled in accordance with the GMFCS, which offers a way to categorize patients with cerebral palsy into one of five levels secondary to their functional abilities. Children in level V have trouble managing their head and trunk posture in most postures and in attaining any voluntary control of movement, while children in level I can complete all the activities of their age-matched peers, but with some difficulty with speed, balance, and coordination [36].

Children with CP between the ages of 3 and 18 years-old who had a GMFCS level of I to V, the capacity to express discomfort or suffering, and the comprehension of straightforward instructions were included in the study.

Medical issues that would interfere with the locomotor training or physical restrictions on utilizing the robotic device were among the exclusion criteria. Moreover, children showing severe lower-extremity muscle contractures, hip instability or subluxation, recent Botulinum toxin-A (BTX-A) injections to the lower limbs, an uncontrolled seizure disorder, open skin lesions, and a vascular disorder of the lower limbs were excluded from the study.

Ten children with CP were enrolled in this study between November 2021 and April 2022 at the Neurorehabilitation Unit “Gli Angeli di Padre Pio” of the Padre Pio Foundation and Rehabilitation Centers, San Giovanni Rotondo, Foggia, Italy. After two of them tested positive for SARS-CoV-2 during the training, they withdrew, leaving the final sample to be made up of eight children (age: 9.88 ± 4.73 years) affected by spastic CP subtype (seven of them were bilaterally affected). In agreement with the GMFCS classification, one participant was classified as level I, one in level III, five in level IV, and one in level V.

### 2.2. Clinical Evaluation

The participants underwent a total of 12 RAGT sessions in one month (three times per week) lasting 30 min. The Lokomat’s biofeedback was used to administer RAGT. Notably, the individuals received conventional therapy in addition to the RAGT. To assess the participants’ changes in their motor skills, the Gross Motor Function Measure-88 (GMFM-88) was administered before (T0) and after the training (T2). This test measures 88 movements across five dimensions: lying down and rolling over; sitting; crawling and kneeling; standing; and walking, running, and jumping [37].

The Shapiro–Wilk normality test was used to determine whether the distribution of the clinical data scores was normal. The Wilcoxon signed-rank test was used to compare the results from the two sessions (i.e., T0 and T2) in order to assess the change in muscle tone and degree of spasticity in response to RAGT because the data did not conform to the assumption of normality.

### 2.3. Experimental Design

**Figure 1:**
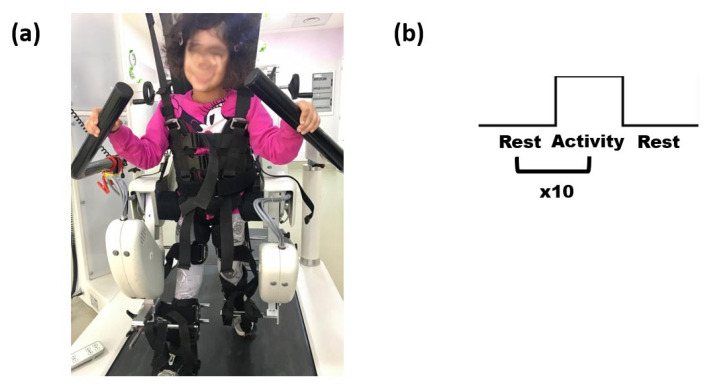
(**a**) Representative participant tethered to the Lokomat. Once the participants were harnessed on the exoskeleton for the robotic therapy, the thermal camera was placed to focus on the participant’s face. (**b**) Representation of the block designed paradigm employed for the experimental sessions. The block design paradigm allowed to administer the RAGT in an ecological way and to evaluate the temperature changes during the walking phases with respect to a rest period. Notably, 10 blocks were considered in the experiment; hence, 10 temporal windows were analyzed for each patient.

The RAGT experimental procedure was given to the subjects once they were tethered to the Lokomat (Figure 1a). The children were specifically instructed to move actively during the RAGT for 30 s before being invited to relax for another 30 s using a block paradigm. Ten blocks, as seen in Figure 1b, made up the paradigm.

### 2.4. IRT Measurements and Data Analysis

The FLIR SC660 (FLIR, Wilsonville, OR, USA) (640 × 480 bolometer FPA, sensitivity/noise equivalent temperature difference: 30 mK @ 30 °C, field of view: 24° 18°) digital thermal infrared camera was used to measure the facial temperature during the first (T0), the sixth (T1), and the twelfth (T2) sessions. The subject was positioned with the camera 60 cm away and pointed directly at his/her face. The frequency rate was set to 10 Hz. The camera was blackbody calibrated to reduce any potential sensor response drift/shift as well as optical artifacts.

The children arrived in the experimental room 15 min before the start of the session, to allow their baseline skin temperature to stabilize. The temperature and humidity of the room were kept stable at 23 °C and 50–60%, respectively, and controlled through a thermostat. The recommendations for thermal measurements made by Ring and Ammer (2012) [38], Diakides, Bronzino, and Peterson (2012) [39], and Merla and Romani (2006) [40] were followed.

**Figure 2:**
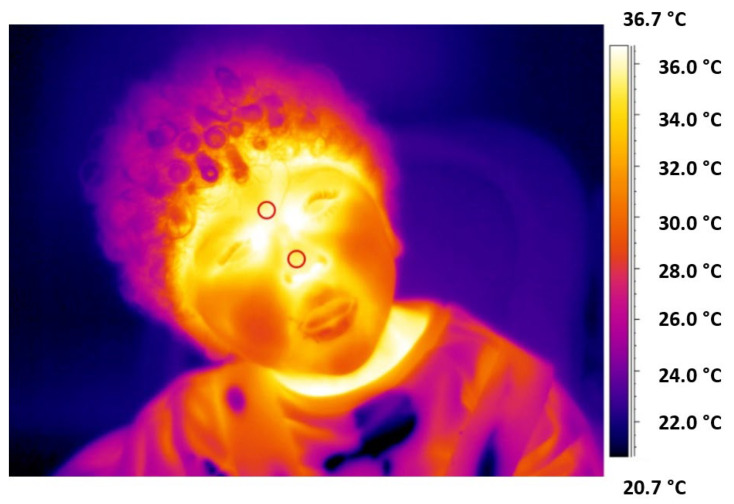
Thermogram of a representative participant with indicated and selcted ROIs over the tip of the nose and the corrugator. Thanks to a tracking algorithm, the ROIs were followed across the frames of the infrared video recorded, allowing to dynamically monitor the temperature time course of the selected ROIs. This approach allowed to evaluate linear and non-linear metrics indicative of the psychophysiological state of the participants.

Regarding the IRT signals data analysis, visual inspection was used to assess the captured thermal signals’ quality first; no video was disallowed. Two regions of interest (ROI) were chosen on the nose tip and forehead (corrugator) of each subject (Figure 2). These regions were selected; hence, they are highly indicative of the autonomic nervous system activity [25]. The position of these ROIs was tracked across all the frames of the thermal video employing a tracking algorithm [41]. The corrupted samples were replaced with the mean value of six samples taken before and after the motion period when the tracking algorithm failed (for example, due to a head rotation that was too wide). Notably, children were instructed to watch straight in front of them during the therapy; hence, only 12% of the total frames needed to be corrected. Moreover, the Hampel filter was used to remove possible motion residual artifacts from the temperature time course. In detail, the Hampel filter is a reliable outlier detector that uses median absolute deviation. The median and standard deviation are determined for each sample of the signal utilizing all nearby values within a time window (in this study, 15 s). The point of interest is classified as an outlier and replaced by the median value if it is more than *n* (*n* = 2 in this case) standard deviations from the median. The ROIs placement on the face of a representative participant is reported in Figure 2.

Subsequently, the following features were computed from the thermal signals for each ROI:

1. Mean value (*MeanTemp*)—average value of the thermal signal *T* over time (i.e., *N* samples) defined as:
  (1) $$MeanTemp=\frac{1}{N}\overset{N}{\underset{i=1}{\sum }}Ti$$
2. Standard deviation (*STD*)—standard deviation of the thermal signal *T* overtime (i.e., *N* samples) defined as:
  (2) $$STD=\sqrt{\frac{1}{N-1}\overset{N}{\underset{i=1}{\sum }}{\left(T_{i}-MeanTemp\right)}^{2}}$$
3. Sample Entropy (*SampEn*): defined as the negative natural logarithm of the conditional probability that signals that the subseries of length *m* (pattern length) that match pointwise within a tolerance *r* (similarity factor) also match at the *m* + 1 point. *SampEn* of a time series {*t*_1_,……,*t_N_*} of length *N* is computed employing the following set of equations:
  (3) $$SampEn\left(m,r,N\right)=-\ln\left[\frac{U^{m+1}\left(r\right)}{U^{m}\left(r\right)}\right]\;U^{m}\left(r\right)={\left[N-m\tau \right]}^{-1}\overset{N-m\tau }{\underset{i=1}{\sum }}C_{i}^{m}\left(r\right)\;C_{i}^{m}\left(r\right)=\frac{B_{i}}{N-\left(m+1\right)\tau }\;B_{i}=number\;of\;j\;where\;d\left|T_{i},T_{j}\right|\leq r\;T_{i}=\left(t_{i},t_{i+\tau },\ldots ,t_{i+\left(m-1\right)\tau }\right)\;T_{j}=\left(t_{j},t_{j+\tau },\ldots ,t_{j+\left(m-1\right)\tau }\right)\;i\leq j\leq N-m\tau ,\;j\neq i$$

In this study, it has been considered that *m* = 2 and *r* = 0.2 ∙ SD of the signal. These parameters are commonly employed for complexity analysis of biological signals and they were chosen in accordance with [42,43].

These metrics were evaluated for each block and the average across the blocks was used for further statistical analysis.

The one-way repeated measure ANOVA (RM-ANOVA) has been employed in order to evaluate statistical differences between the thermal features at T0, T1, and T2, considering the temporal session as within factor. Then, multiple comparisons (paired *t*-test) were conducted to determine which temporal recordings offered statistical differences. The statistics were then corrected for multiple comparisons (false discovery rate, FDR) to eliminate false positives.

## 3. Results

Concerning the clinical scales evaluation, the GMFM-88 showed a significant change between T0 and T2 (T0 vs. T2, z = −2.524; *p* = 0.008). The modification of the GMFM-88 for each participant is reported in Table 1. Notably, the GMFCS level is specified for each participant to highlight a possible dependance of the rehabilitation outcome improvement from the GMFCS level.

**Figure 3:**
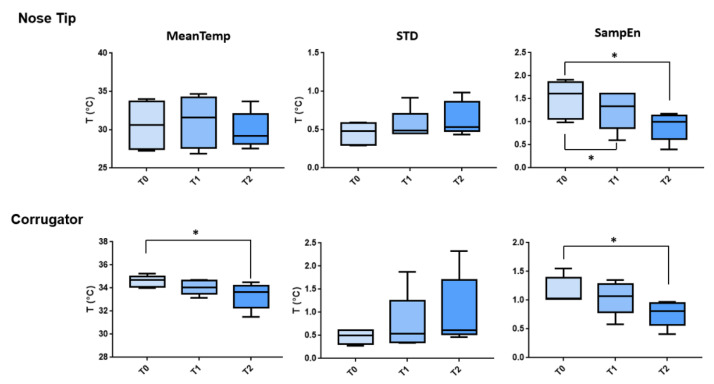
Boxplot associated with the thermal features evaluated for the nose tip and corrugator across the experimental sessions (i.e., T0, T1, and T2). The significant comparisons are highlighted in the figure with the asterisks (*).

A statistical analysis revealed that the patients’ emotional state changed during the course of the many sessions. A significant difference was reported for the SampEn (F (1.477, 5.907) = 6.888; *p* = 0.033) and the mean value (F (1.425, 5.7) = 5.88; *p* = 0.047) specifically with reference to the corrugator. The SampEn revealed significant differences with regard to the tip of the nose (F (1.134, 4.536) = 11.5; *p* = 0.041). A boxplot showing the modifications of the thermal features across the sections is reported in Figure 3.

Multiple comparisons, FDR corrected, revealed a significant difference for the SampEn computed over the nose tip across all sessions, but only between T0 and T2 for the metrics evaluated on the corrugator temperature time course. The multiple comparisons results are summarized in Table 2.

## 4. Discussion

The aim of this study was to assess the psychophysiological correlates associated with RAGT in children with CP using IRT. The rationale of the study relies on previous findings demonstrating that children should actively participate in the therapeutic process to maximize the benefits of rehabilitation [44,45]. In fact, children’s motivation, interest in, and perseverance with rehabilitation activities, as well as the quality of the relationship developed with the therapist, may have an impact on therapy engagement [46]. The sociocultural, physical, and temporal circumstances in which therapy is provided may potentially help or hurt children’s motivation to participate in it [47]. Of note, in the literature it is known that children with CP exhibit less mastery motivation than their typically developed counterparts [48,49]. Recent research suggests that customized therapy outcomes are better for children with unilateral CP who exhibit higher task perseverance in age-appropriate activities (as reported by their parents) both immediately after treatment and 26 weeks post-intervention [50,51]. In addition, Harniess et al. showed that the underlying intervention resources provided by particular techniques (e.g., coaching pedagogy) are related to the positive parent reasoning mechanisms of trust, belief, sense of control, perceived feasibility of home program delivery, and finally, motivation. These reactions are the starting points for engagement outcomes such as improved parental adherence and self-efficacy. Parental self-efficacy is significant because it can start a process of change that improves parental confidence and anxiety [52].

However, it should be highlighted that these mentioned studies evaluate the engagement of the children to therapy through interviews to the children and to their parents, but they do not provide a physiological correlate of the psychophysiological condition of the child during the treatment. In fact, techniques such as IRT can provide information on the affective states of children which are related to the participation to therapy.

To the best of the authors’ knowledge, this is the first study investigating the autonomic implications of RAGT in CP patients. To this aim, IRT has been demonstrated to be highly accepted by the children during testing thanks to its contactless and non-invasive features.

The findings demonstrated a modulation of the thermal response across the sessions. Particularly, a decrease of the SampEn evaluated on the temperature time course of both the corrugator and the tip of the nose was found, whereas a significant decrease of the MeanTemp of the corrugator was assessed.

The temperature decrease assessed on the forehead could be associated with the activation of the sympathetic system, whose activation is associated also with attention and concentration [53]. This interpretation is confirmed also by the SampEn results. In fact, it is well known that a lower signal complexity is correlated with an increase in the low-frequency component (or a decrease in the high-frequency component) [54]. An increase low frequency component of thermal signal, which highly depends on the superficial microcirculation, is indicative of a sympathetic predominance over parasympathetic activity [55]. Hence, it could be deduced that the decreased SampEn is ascribed to a prevalent sympathetic activity.

Concerning this complexity analysis, SampEn’s embedding dimension (m) and time delay (τ) need to often be optimized based on the dynamic nature of the target signal. However, there is a practical limitation based on the sampling frequency and overall recording time (total number of samples). Richman and Moorman specifically proposed and tested the SampEn metric, which limits the m value based on the total number of samples of the target signal to be contained in the range of 10–20 m [56]. Furthermore, it was demonstrated that choosing m = 2 is preferable to m = 1 since it enables more accurate reconstruction of the process’s joint probabilistic dynamics [57]. In this work, the total number of samples for each phase allowed us to use a maximum of m = 2 combined with τ = 1, despite the possibility that various values of m and τ can provide stronger sensitivity to changes in IRT signal complexity. Longer blocks could be employed in further studies to examine various values of m and τ.

These findings suggest an improvement of the engagement of the children during the different RAGT sessions. Notably, the multiple comparisons analysis assessed significant differences, FDR corrected, mostly between T0 and T2. Hence, the results suggest that modulations of the psychophysiological state occur in children after several sessions. However, further studies should investigate different RAGT protocols (e.g., frequency of the treatment and duration of each session) to assess which induces the best benefits to the children’s engagement.

Furthermore, it should be highlighted that the sympathetic activity is also related to negative affective states, such as stress and perception of danger. Hence, further studies should be performed to investigate the affective state of the CP children, developing models of affective computing suitable for this kind of pathology. An interesting approach could rely on multimodal monitoring of the autonomic activity of the children during RAGT (e.g., heart rate variability and galvanic skin response), paying attention to preserving the ergonomic state of the patients during the therapy.

However, the significant increase in the GMFM-88 scores after the whole sessions could suggest that the increased sympathetic activity assessed in this study could be related to an increase of the engagement of the children to the therapy. This result is consistent with other research showing the RAGT’s advantages for diplegic children with CP. For instance, Wallard et al. found that RAGT therapy administered through Hocoma Lokomat significantly improved the kinematic data of the full body in the sagittal and frontal planes as well as the gross motor function measure test, demonstrating the value of RAGT in enhancing balance control in gait [58]. Van Kammen and colleagues also discovered benefits connected to the RAGT. They showed that walking with the Lokomat decreases muscular activity in kids with CP, but changing guidance or BWS typically has little effect on amplitude [59].

Notably, all the participants exhibited an improvement of the GMFM-88 scores, and, specifically, the highest modifications were associated with the lower GMFCS levels. This finding could suggest that RAGT is more effective in patients with severely compromised motor abilities. Importantly, the GMFM-88 was preferred with respect to other clinical scales because it investigates several motor abilities, not only related to the standing or gait activity. In fact, the five dimensions evaluated by the GMFM-88 are: lying and rolling (GMFM-A); sitting (GMFM-B); crawling and kneeling (GMFM-C); standing (GMFM-D); and walking, running, and jumping (GMFM-E). Since most of the children considered in this study were not able to walk autonomously (seven out of eight participants), the GMFM-88 was adopted to evaluate the rehabilitation outcome improvements. However, further studies should be performed employing other clinical scales to assess the rehabilitation outcomes. For instance, the modified Ashworth scale (MAS) created by Bryan Ashworth as a tool for evaluating spasticity is suitable for this typology of patients. The original Ashworth scale was a five-point numerical scale, with 0 denoting no resistance and 4 denoting a rigid limb in flexion or extension, and it was used to grade spasticity. However, in order to boost sensitivity, Bohannon and Smith added 1+ to the Ashworth scale. Since there is no increase in muscle tone, MAS ranges from 0 (no rigidity in flexion or extension) to 4 [60]. These findings may foster the use of IRT in clinical settings with the goal of determining the participation of patients to therapy. This would enable the treatment to be tailored to the psychophysiological condition of the patient, enhancing its efficacy. These results might also encourage the development of shared rules for RAGT administration. Currently, there are no standardized dosages or RAGT standards for CP. In the literature, there are only two reviews that describe, respectively, the robotic treatment of 486 CP patients throughout 17 trials [61] and 217 patients across 10 studies [62] (Carvalho et al., 2017). These reviews demonstrate that most studies focus more on children with CP classed as I-IV. Furthermore, the two surveys report a heterogeneity in the selection of treatment protocols, whose lengths range from 30 to 60 min. According to Sarhan et al., sessions are repeated for 2–6 weeks, up to a maximum of 10 weeks, and they range from two to five per week [63]. Then, in order to eliminate clinical approach heterogeneity and optimize the neurological, motor, and psychophysiological advantages of the therapy, it would be advisable to use IRT in the clinical setting to standardize the RAGT protocols and to increase the engagement of children with CP.

It should be emphasized that the protocol did not apply to uncooperative participants or to children who could not understand the augmentative feedback displayed on Lokomat’s screen. Future research should focus on determining whether intense RAGT can aid children with severe cognitive deficits who are typically excluded from robotic rehabilitation procedures because of their uncooperativeness.

Finally, it should be stressed that this approach could be relevant to patients that are not able to speak and provide feedback during the procedure (e.g., children affected by dysarthria). In fact, CP is often accompanied by several impairments such as behavior disorders and inability to talk. This study could pave the way for further experimentations aiming to monitor and predict the effectiveness of the rehabilitation also in other pathologies with the goal to administrate the more suitable and acceptable therapy for each patient.

## 5. Conclusions

This research reports about the capability of employing IRT during RAGT in order to evaluate the psychophysiological state of CP children during the therapy. The findings demonstrated that CP patients enhance their attention, concentration, and motor control during the therapy across the different RAGT sessions. This method can be utilized to customize clinical treatments, enhancing the success of CP children’s rehabilitation and, as a result, their quality of life. This work may open the way for additional research targeted at tracking and forecasting the efficacy of rehabilitation also in other disorders with the goal of providing each patient with the most appropriate therapy.

## Figures and Tables

**Table 1:** GMFM-88 and GMFCS level improvements for each participant between T0 and T2 associated with RAGT.

Subj	GMFCS Level (T0)	GMFCS Level (T2)	GMFM-88 (T0)	GMFM-88 (T2)	Delta	Delta (%)
1	III	III	32.10	42.00	9.9	30.8
2	IV	IV	11.10	16.10	3	45.0
3	I	I	47.20	49.20	2	4.1
4	IV	III	17.20	25.50	8.3	48.3
5	IV	III	30.00	36.30	6.3	21.0
6	IV	III	16.00	24.30	8.3	51.9
7	V	IV	15.50	22.40	6.9	44.5
8	IV	III	12.40	16.30	3.9	31.4

**Table 2:** Multiple comparisons (paired *t*-test) related to the RM-ANOVA. In the table, only significant comparisons are shown.

ROI	Metric	Comparison	t-Stat	Adjusted *p*-Value
Nose Tip	SampEn	T0 vs. T1	3.751	0.0426
	SampEn	T0 vs. T2	3.582	0.0492
Corrugator	MeanTemp	T0 vs. T2	3.672	0.0455
	SampEn	T0 vs T2	4.506	0.0234

## Data Availability

The data presented in this study are available upon request from the corresponding author. The data are not publicly available due to privacy issues.
